# How does healthcare worker hand hygiene behaviour impact upon the transmission of MRSA between patients?: an analysis using a Monte Carlo model

**DOI:** 10.1186/1471-2334-9-64

**Published:** 2009-05-15

**Authors:** Clive B Beggs, Simon J Shepherd, Kevin G Kerr

**Affiliations:** 1 School of Engineering, Design and Technology, University of Bradford, Bradford BD7 1DP, UK; 2 Departments of Microbiology Harrogate and District NHS Hospital Health Care Trust, Harrogate District Hospital, Lancaster Park Road, Harrogate HG2 7SX, UK

## Abstract

**Background:**

Good hand hygiene has for many years been considered to be the most important measure that can be applied to prevent the spread of healthcare-associated infection (HAI). Continuous emphasis on this intervention has lead to the widespread opinion that HAI rates can be greatly reduced by increased hand hygiene compliance alone. However, this assumes that the effectiveness of hand hygiene is not constrained by other factors and that improved compliance in excess of a given level, in itself, will result in a commensurate reduction in the incidence of HAI. However, there is evidence that the law of diminishing returns applies to hand hygiene, with the greatest benefits occurring in the first 20% or so of compliance. While this raises intriguing questions about the extent to which increasing compliance alone can further reduce rates of HAI, analysis of this subject has been hampered by a lack of quantifiable data relating to the risk of transmission between patients on wards.

**Methods:**

In order to gain a greater understanding of the transmission of infection between patients via the hands of healthcare workers (HCWs), we constructed a stochastic Monte Carlo model to simulate the spread of methicillin-resistant *Staphylococcus aureus *(MRSA) between patients. We used the model to calculate the risk of transmission occurring, firstly between two patients in adjacent beds, and then between patients in a four-bedded bay. The aim of the study was to quantify the probability of transmission under a variety of conditions and thus to gain an understanding of the contribution made by the various factors which influence transmission.

**Results:**

The study revealed that on a four-bedded bay, the average probability of transmitting an infection by the handborne route is generally low (i.e. in the region 0.002 – 0.013 depending on the hand hygiene behaviour of HCWs and other factors). However, because transmission is strongly influenced by stochastic events, it is the frequency with which 'high-risk events' occur, rather than average probability, that governs whether or not transmission will take place. The study revealed that increased hand hygiene compliance has a dramatic impact on the frequency with which 'high-risk events' occur. As compliance increases, so the rate at which 'high-risk events' occur, rapidly decreases, until a point is reached, beyond which, further hand hygiene is unlikely to yield any greater benefit.

**Conclusion:**

The findings of the study confirm those of other researchers and suggest that the greatest benefits derived from hand hygiene occur as a result of the first tranche of compliance, with higher levels (>50%) of hand hygiene events yielding only marginal benefits. This suggests that in most situations relatively little benefit is accrued from seeking to achieve very high levels of hand hygiene compliance.

## Background

Good hand hygiene has for many years been considered the single most important measure that can be applied to prevent the spread of healthcare-associated infection (HAI) [1]. Through regular cleansing of hands, healthcare workers (HCWs) reduce the risk to transmitting pathogens between patients and thus reduce the risk of exogenously-acquired infection. This has led to the widespread opinion that HAI rates can be greatly reduced by increased hand hygiene compliance alone [2]. However, while it is undoubtedly the case that improved hand hygiene is beneficial [1,3], there is evidence to suggest that the law of diminishing returns applies to hand hygiene, with the greatest benefits occurring in the first tranche of compliance [4,5]. Recently we used a deterministic Ross-Macdonald model of a medical ward [6], to demonstrate that, in most situations, little benefit is accrued from very high levels of hand hygiene. While this study yielded insights into the spread of infection through imperfect hand hygiene practised by HCWs in hospital settings, it was hampered by the fact that our model was deterministic in nature and unable to take into account the chance events that frequently occur in such environments. Therefore in order to gain a greater understanding of the transmission of infection between patients via the hands of HCWs, we constructed a novel stochastic Monte Carlo model. We used this model to simulate the spread of methicillin-resistant *Staphylococcus aureus *(MRSA) between the patients in adjacent beds, with the aim of understanding the contribution of the various factors which influence transmission.

## Methods

A stochastic model to analyse the transmission of MRSA between patients via the hands of HCWs was constructed using Microsoft Excel. The model used Monte Carlo methodology to simulate the transmission of infection between two adjacent patients, denoted A and B. In the model it was assumed that:

• Patient A is the actual source *of *MRSA.

• Patient A is continuously, and not intermittently, colonized with the bacterium.

• The HCWs movements are in one direction only (i.e. from A to B).

• The transmission of MRSA is caused only by contact with the transiently colonized hands of the HCW.

• No gloves or other personal protective equipment are used by HCWs.

• Contacts between the transiently colonized HCW and the uncolonized patient have a given probability of colonizing the patient, which is termed the HCW-to-patient transmissibility, *p*.

• The HCW acquires transient hand-contamination only by touching the colonized patient. All such contacts between the uncolonized HCW and colonized patient have a given probability of colonizing the carer, which is termed the patient-to-HCW transmissibility, *p*'.

• MRSA can only be removed from the hands of HCWs by hand hygiene, with the level of compliance (using the standard that hands are cleansed before and after direct patient contact) being γ.

• The efficacy of the hand hygiene process, λ, is less than 100% meaning that not all MRSA bacteria are eliminated.

• The values of *p*, *p*', γ and λ are normally distributed.

For ease of computation, simulations were conducted in batches of 100. In each simulation batch it was assumed that a HCW made 100 journeys from Patient A to Patient B, and that on each occasion physical contact was made between the HCW and the two patients. The number of hand hygiene events that occurred during the various movements was determined using a normally distributed random number generator. Similarly, the precise values of *p*, *p*', γ and λ were determined using a normally distributed random number generator. Each batch was then repeated 1000 times, making a total of 100 000 individual simulations in all.

While mean values and standard deviations for *p*, *p*', γ and λ are specified by the user, to ensure stochasticity a random number generator was used in the model to determine the precise values of each variable during any given batch simulation. So for example, if hand hygiene compliance, γ, is 0.4, then in each simulation batch the model will randomly allocate 40 hand hygiene events to the 100 HCW journeys. A similar approach is taken for *p *and *p*', with the number contamination events being stochastically determined and then randomly allocated in a discrete manner to the 100 HCW journeys. For any given journey, transmission of infection from one patient to another is deemed to occur when: contamination of the HCW's hands occurs following contact with Patient A, followed by contamination of Patient B via contact with the uncleansed hands of the HCW.

Transmission can also occur when the efficacy of the hand hygiene process, λ, is less than 1.0, with the precise risk being determined by applying the coefficient (1-λ) to the probability score for the individual journey.

Having created the model for transmission of MRSA from Patient A to Patient B, we then placed both these patients in a four bedded bay (where they were joined by patients C and D) and created a random walk model to calculate the risk of transmission from Patient A (the source of MRSA) to patients B, C or D (all of whom were not colonised at the outset of the experiment). This model utilized the same Monte Carlo methodology as that described above and used a random number generator to determine the various journeys made the HCWs (e.g. from Patient A to Patient D; from Patient B to Patient C; etc.). Only those HCW journeys which originated with Patient A were deemed to carry any risk. All other journeys, including those that ended at Patient A, were deemed to carry no risk.

### Model Scenarios

Because we wanted to evaluate the impact of placing an MRSA carrier in a ward it was assumed that Patient A was continuously colonized The default mean values for *p*' and *p *were assumed to be 0.4 and 0.1, respectively, similar to those used by Austin *et al *[7] (see Table 1). This implies that the HCW is four times more likely to contaminate their hands through contact with a colonized patient than he/she is to colonize a subsequent patient.

**Table 1:** Default values used in stochastic model

Parameter	Meaning	Mean Value	Standard Deviation
γ	Hand hygiene compliance	0.40	0.10
*p*	HCW-patient transmission probability (i.e. transmissibility)	0.10	0.10
*p*'	Patient-HCW transmission probability (i.e. transmissibility)	0.40	0.20
λ	Efficacy hand hygiene process	0.83	0.10

In our study we modelled the effect of varying hand hygiene compliance, γ, (i.e. γ = 0.0, 0.1, 0.2 ... 1.0) on the transmission of MRSA, firstly from Patient A to Patient B, and then from Patient A to patients B, C and D. In this analysis it was assumed that the hand hygiene efficacy was either 58% or 83% – as reported by Girou *et al *[8] for HCWs in a clinical setting using antibacterial soap and an alcohol-based solution, respectively. Finally, we also evaluated the impact of varying the value of *p*'.

## Results

**Figure 1:**
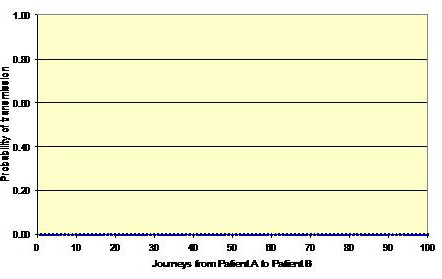
**Probability of transmission between Patient A and Patient B for a batch of 100 HCW journeys**. No risk of transmission – the situation that occurs in many simulations.

**Figure 2:**
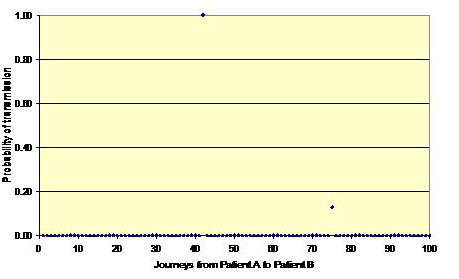
**Probability of transmission between Patient A and Patient B for a batch of 100 HCW journeys**. Low risk of transmission – the situation that occurs in some simulations.

**Figure 3:**
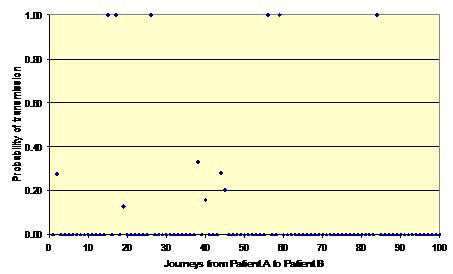
**Probability of transmission between Patient A and Patient B for a batch of 100 HCW journeys**. High risk of transmission – the situation that occurs in a few simulations.

Figures 1, 2 and 3 shows three simulation outcomes (chosen at random for illustrative purposes) arising from the same input data (i.e. mean values for *p*, *p*', γ and λ of 0.40, 0.10, 0.40 and 0.83, respectively). Figure 1 represents a batch where no transmission occurs between patients A and B and is the outcome that arises most often. Figure 2 shows a situation where some transmission occurs, but the risk is still relatively low, and Figure 3 represents a potential outbreak situation, where the risk of transmission is high – a situation that occurs very infrequently. From this it can be seen that on most occasions when HCWs move from one patient to another, there is no risk to the latter. However, on occasions chance events conspire to create a situation where the risk of transmission can be relatively high. For example, in Figure 3 it can be seen that twelve of the movements from Patient A to Patient B pose a risk. The intervention of the hand hygiene process is clearly visible for six of these interpatient movements in Figure 3, whereas on six other occasions the fact that the HCW did not practice hand hygiene is evident (i.e. the probability of transmission equals 1.0). On the occasions where the probability of transmission is less than one, but greater than zero, this indicates that although the HCW undertook hands hygiene, the efficacy of the cleansing process was less than 1.0.

The results of varying hand hygiene compliance for HCWs using both antibacterial soap (γ = 0.58) and an alcohol-based solution (γ = 0.83), under the default conditions, are shown in Table 2. From this it can be seen that:

**Table 2:** Results of 100 000 stochastic HCW journeys from Patient A to Patient B for various levels of hand hygiene compliance, assuming mean values for *p *and *p*' of 0.10 and 0.40, respectively

Hand hygiene compliance	Average probability of transmission between patients A and B (Alcohol rub: γ = 0.83 with st. dev. = 0.1)	Standard deviation on probability data (Alcohol rub: γ = 0.83)	Average probability of transmission between patients A and B (Soap: γ = 0.58 with st. dev. = 0.1)	Standard deviation on probability data (Soap: γ = 0.58)
0	0.0403	0.0049	0.0425	0.0039
10	0.0368	0.0056	0.0381	0.0039
20	0.0348	0.0045	0.0357	0.0039
30	0.0318	0.0035	0.0318	0.0023
40	0.0266	0.0041	0.0339	0.0039
50	0.0247	0.0041	0.0307	0.0039
60	0.0212	0.0018	0.0268	0.0039
70	0.0174	0.0022	0.0246	0.0015
80	0.0153	0.0015	0.0236	0.0031
90	0.0108	0.0012	0.0204	0.0021
100	0.0082	0.0011	0.0165	0.0021

• Even with no hand hygiene compliance at all, the average risk of transmission from Patient A to Patient B is only about 4%) – implying that most journeys made by HCWs pose no risk at all.

• At lower levels of hand hygiene compliance little benefit is gained from using the alcohol hand rub, despite the fact that the mean efficacy of the alcohol rub is 0.83, compared with 0.58 for the antibacterial soap (as determined by *Girou *et al [8]). However as compliance increases, so the alcohol rub out-performs the soap.

• Although an increase in hand hygiene compliance generally reduces the risk of transmission between adjacent patients, because of stochasticity (i.e. chance events), increased hand hygiene compliance does not always result in a lower risk of transmission between patients.

**Figure 4:**
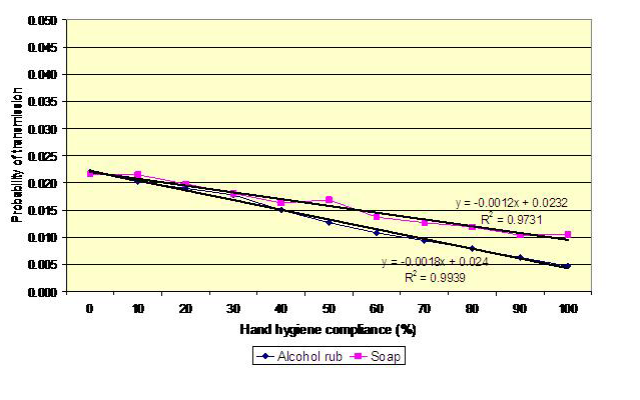
**Impact of hand hygiene compliance on the probability of transmission between patients A and B**. Mean values for *p *and *p*' of 0.10 and 0.20, respectively are assumed; hand hygiene efficacy of soap and alcohol rub is 0.58 and 0.83, respectively.

**Figure 5:**
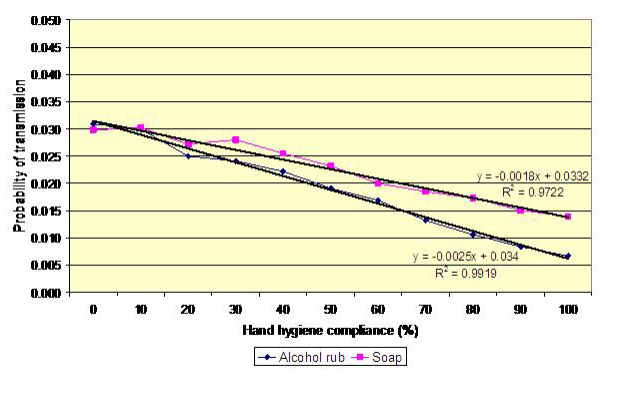
**Impact of hand hygiene compliance on the probability of transmission between patients A and B**. Mean values for *p *and *p*' of 0.10 and 0.30, respectively are assumed; hand hygiene efficacy of soap and alcohol rub as before.

**Figure 6:**
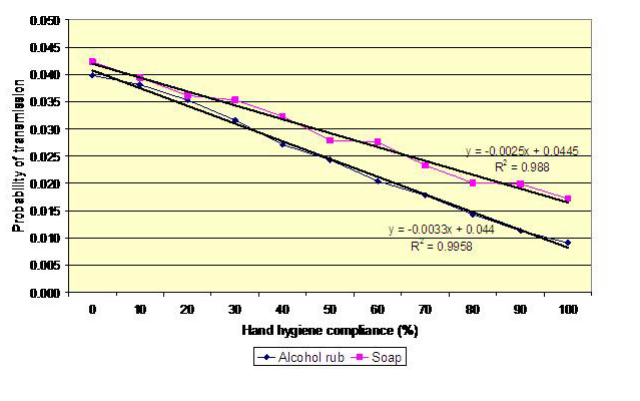
**Impact of hand hygiene compliance on the probability of transmission between patients A and B**. Mean values for *p *and *p*' of 0.10 and 0.40, respectively are assumed; hand hygiene efficacy of soap and alcohol rub as before.

Figures 4, 5 and 6 present the results of analysis similar to that in Table 2, for transmission between patients A and B and for varying values of *p*'(i.e. *p*' = 0.2, 0.3 and 0.4). This reveals *p*' to be particularly influential; something which is not surprising, given than contamination of the hands of HCWs is the key event which initiates the transmission of infection – if a HCW's hands are uncontaminated then no infection can be transmitted via the hands. When the probability that a HCW worker will contaminate his/her hands, *p*', is high, the benefits derived from increased hand hygiene compliance are much greater than when *p*' is small. Notwithstanding this, for all values of *p*', the benefits derived from increased hand hygiene compliance are relatively small compared with the effort required to secure that additional compliance. For example, from Figure 4 it can be seen that a 20% increase in hand hygiene compliance equates to a reduction in the risk of transmission of less than 0.5%, assuming that an alcohol-based hand gel is used. If soap is used, then the reduction is even less.

**Figure 7:**
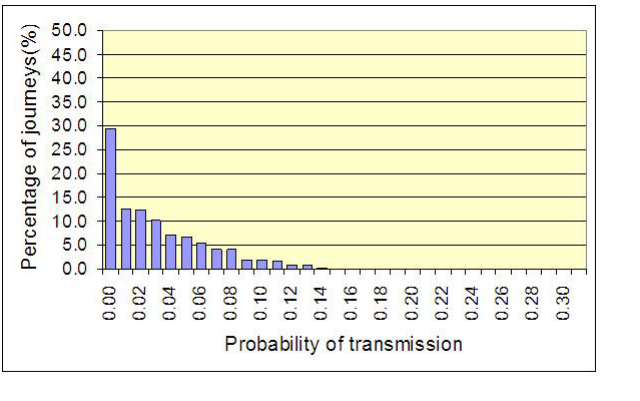
**Frequency distribution of the probability of transmission between Patient A and Patient B for a 1000 batch simulations**. Mean values for *p*, *p*', γ and λ of 0.10, 0.40, 0.40 and 0.83, respectively are assumed).

**Figure 8:**
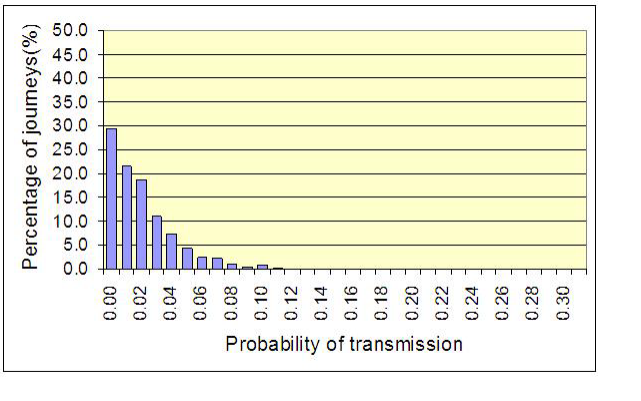
**Frequency distribution of the probability of transmission between Patient A and Patient B for a 1000 batch simulations**. Mean values for *p*, *p*', γ and λ of 0.10, 0.40, 0.70 and 0.83, respectively are assumed.

These results are the average probabilities generated by the model. While they give a good indication of the quantifiable benefits of hand hygiene, they give little indication of variations that occur due to random events. However chance events are of great importance and it is critical to take into account those rare events, such as those shown in Figure 3, which may lead to an outbreak of MRSA infection. Figure 7 shows the frequency distribution for the results of a 1000 batch simulations and is typical for the default condition. From this it can be seen that 29.4% of batch simulations resulted in no risk of transmission at all. Indeed, 42.1% of simulations resulted in a risk of transmission <2% between patients A and B. However, 4.2% of simulations resulted in a risk of transmission >10%, with one simulation producing a risk of 24.0%. By comparison Figure 8 shows the frequency distribution when hand hygiene compliance is 70% (i.e. 30% above the default condition). From this it can be seen that the improvement in compliance 'shortens' the frequency curve, so that now 51.0% of simulations resulted in a risk of transmission <2%, and only 0.5% of simulations result in a risk of transmission >10%, with the highest risk recorded is now 18.0%.

From the analysis above it can be concluded that, as HCW hand hygiene compliance increases, so the number of high risk events reduces. This phenomenon is quantified in Table 3, which shows the probability frequency distribution for the default condition over a range of compliance levels.

**Table 3:** Frequency distribution of the probability of transmission between Patient A and Patient B for a range of hand hygiene compliance levels (assuming mean values for *p*, *p*', γ and λ of 0.10, 0.40, 0.40 and 0.83, respectively).

Hand hygiene compliance	Simulations resulting in a risk >1% [%]	Simulations resulting in a risk >5% [%]	Simulations resulting in a risk >10% [%]	Simulations resulting in a risk >15% [%]	Simulations resulting in a risk >20% [%]
0	62.3	29.1	8.3	2.5	1.0

10	63.2	30.6	7.9	1.9	0.6

20	64.5	28.4	7.6	0.8	0.3

30	61.8	21.8	4.6	0.5	0.0

40	60.4	21.0	3.9	0.3	0.0

50	60.3	17.7	2.3	0.1	0.0

60	55.1	12.8	1.5	0.2	0.1

70	50.3	8.3	0.1	0.0	0.0

80	47.7	5.5	0.0	0.0	0.0

90	39.8	1.7	0.0	0.0	0.0

100	31.3	0.7	0.0	0.0	0.0

### Four Bedded Bay

**Figure 9:**
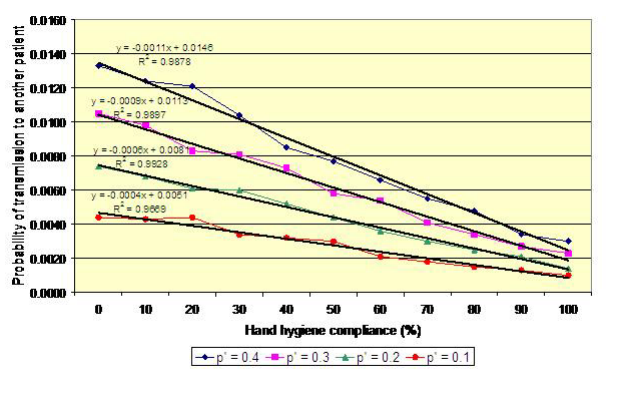
**Impact of hand hygiene compliance on the probability of transmission from Patient A to another patient on a four-bedded bay**. A mean value for *p *of 0.10 and a hand hygiene efficacy of 83% for alcohol rub is assumed.

Colonized patients may be placed in ward bays containing more than one other patient. This may occur for example before colonization status is known or because of insufficiency of isolation rooms. Therefore, in order to gain a greater understanding of the risks posed by placing a colonized patient in a multi-bed area, we simulated MRSA transmission in a four-bedded bay, containing patients A, B, C and D, using a random walk Monte Carlo model. The results of this analysis are presented in Figure 9, which shows the risk of colonisation for another patient cared for in the same bay as Patient A (i.e. the continuous carrier), assuming that alcohol rub is used by the HCWs attending to all patients in the bay. Thus, under the default condition, when hand hygiene compliance is 40%, each journey made by a HCW to, say, Patient B, involves an average transmission risk of about 0.9% (i.e. on average one journey in 111 journeys results in Patient B becoming colonized). From Figure 9 it can be seen that the curves are similar in shape and slope to those in figures 4, 5 and 6. However, the risk of transmission to patients B, C and D is only a third of that shown in figures 4, 5 and 6. This is because only a third of the HCW journeys to patients B, C and D originate with Patient A – those journeys that do not involve Patient A pose no risk at all. Consequently, the risk that a HCW will transmit MRSA on any given journey between two patients in the bay is very small indeed. Of course, as hand hygiene compliance increases so the risk of transmission decreases. However, because most HCW journeys do not involve Patient A, the benefits of increased hand hygiene compliance are very marginal. For example, when *p*' = 0.2, a 20% increase in hand hygiene compliance only reduces the risk of transmission by less than 0.2%.

## Discussion and conclusion

The study presented in this paper is, to our best knowledge, the first to use this methodology systematically quantify the risk of transmission of a MRSA between patients, via the hands of HCWs. Although purely a mathematical study, it sheds new light on the risks associated with the transmission of MRSA on the hands of HCW and has potentially important implications for infection control practice. Foremost amongst these is the observation that most hand hygiene events may not contribute significantly to the control of infection. From Figure 6 it can be seen that for the worst case scenario (i.e. for a HCW travelling from Patient A [the index case] to Patient B, without practising hand hygiene), the risk of MRSA transmission is approximately 4%. Furthermore, when it is considered that most of the journeys made by the HCW on the four bedded bay do not involve Patient A and therefore pose no risk at all, it is clear that most hand hygiene events may be ineffectual. It could be argued that a blanket approach to hand hygiene is justified on the grounds that it is not possible to know at any given time colonisation status of individual patients. While this non-discriminatory approach has some merit, it has the major drawback that a considerable amount of effort may be wasted in the HCW hand hygiene when it is not needed. In short, the potential benefits of increased hand hygiene are diluted amongst all the patients, rather than focused on those patients who are most likely to be colonized or patients who are already known to be colonised or infected and who therefore pose the greatest risk in infection control terms. Consequently, if the staff on a ward manage to increase compliance by, say 20%, the net benefit of all their additional effort is likely to be much less than it might otherwise be, simply because most of hand hygiene events are expended on journeys that present no risk at all. If however, the HCWs could focus the additional '20% compliance' on those patients who are at highest risk of MRSA carriage, then it is much more likely that transmission rates will be reduced. This simple observation highlights the importance of identifying as quickly as possible those patients who are colonized. If colonized patients are not identified, then a 'blanket' approach must remain in place. However, when colonized patients are detected, then extra precautions, including the need for augmented hand hygiene compliance can be implemented. This approach, however, is dependent, on the ability to identify and isolate MRSA-colonized patients as early as possible, as this will give the best chance of minimizing high-risk stochastic transmission events.

From the data presented in figures 4, 5, 6 and 9 it is tempting to assume that the relationship between hand hygiene and the transmission of MRSA is a linear one. This however is not the case. From Figure 1 it can be seen that transmission of MRSA occurs in discrete events, which are stochastic in nature: thus, the greater the number of 'high-risk events' (such as that shown in Figure 3), the greater the likelihood that transmission will occur. Consequently, it is the frequency of the 'high-risk events', rather than average probability, that, in reality, governs whether or not transmission will occur. From figures 7 and 8 and Table 3 it can be seen that, as hand hygiene compliance increases, so the frequency distribution of the probabilities alters and the number of 'high-risk events' dramatically decreases. When hand hygiene compliance is very low, say 10%, 'high-risk events' occur relatively frequently with the result that transmission between patients is likely to occur. However as compliance increases, so the rate at which 'high-risk events' occur, rapidly decreases, until a point is reached, beyond which, further hand hygiene is unlikely to yield any greater benefit. Indeed, as 'high-risk events' become more infrequent, other factors, such as the admission of MRSA-colonized patients onto wards, tend to become more dominant [6], with the result that no amount of additional hand hygiene can reduce ward prevalence rates. This phenomenon may explain the findings of Beggs *et al *[6] and Cooper *et al *[4], that the relationship between hand hygiene compliance and ward prevalence is asymptotic rather than linear. As such, our analysis provides further evidence that the law of diminishing returns applies to hand hygiene. The greatest benefits are derived from the first tranche of compliance, with higher levels (>50%) of hand hygiene yielding only marginal benefits.

Our data also suggest that the risk of MRSA transmission between patients via the hands of HCWs is generally very small. This can be seen in Figure 9 which effectively quantifies the risk that any given patient on a four bedded bay will become colonized following contact with a HCW who has previously attended to another patient. For the worst case scenario, when *p*' = 0.4 and hand hygiene compliance is zero, the risk of transmission is approximately 1.3%. In other words, a patient will become colonized, on average, after about 77 visits from a HCW. However, when the value of *p*' is smaller and hand hygiene compliance is greater, which is generally the case, this risk greatly reduces For example, when *p*' = 0.2 and compliance is 40%, then the risk of transmission is only 0.5% (i.e. one in 200 journeys). This concurs with the findings of Forrester et al [9] who, when modelling transmission of MRSA on a twelve bed intensive care unit (ICU), reported rates similar to ours.

Our data suggest that the risk of transmission by the handborne route appears to be lower than might be expected. This raises important questions as to the extent to which other routes of transmission or reservoirs of infection, contribute to the spread of MRSA in hospital settings. In this respect it is worth noting that widespread environmental contamination can occur from MRSA colonized patients [10-12], and that environmental contamination has been implicated in several outbreaks of MRSA [13-15]. Efforts to combat MRSA and other healthcare-associated have traditionally regarded HCW hand hygiene as the pre-eminent infection control measure. However, although hand hygiene remains a cornerstone of good infection control practice our data suggest that continuous exhortations to healthcare workers to achieve 100% compliance may not yield the expected benefits that such high levels of compliance are intended to produce.

While the subject of this paper is MRSA, the model presented here is generic in nature and can equally be applied to any pathogen transferred between patients on the hands of HCWs, provided that realistic assumptions are made. However, as with all mathematical models, it is important to appreciate the limitations of our methodology, which applies only transmission via the hands of HCWs. No allowances have been made for the application of personal protective equipment such as gloves, or for the presence of environmental reservoirs that might contribute to the transmission of infection. Furthermore the model did not consider that some patients might be at higher risk of transmitting MRSA such as those with pneumonia or heavily colonised wounds. Consequently, the model may only be of limited applicability to pathogens such as *Clostridium difficile *and *Acinetobacter baumannii *where environmental contamination is thought to play an important role in the transmission of infection.

## Competing interests

The authors declare that they have no competing interests.

## Authors' contributions

CBB and SJS designed the study. CBB and SJS constructed the computer model and KGK advised on the clinical aspects of the study. CBB wrote the manuscript with major contributions from other authors. All authors have read and approved the final manuscript.

## Pre-publication history

The pre-publication history for this paper can be accessed here:

http://www.biomedcentral.com/1471-2334/9/64/prepub
